# Supplementary material for: ER+ Breast Cancer Strongly Depends on MCL-1 and BCL-xL Anti-Apoptotic Proteins
Source: Cells. 2021 Jul 2;10(7):1659. doi: 10.3390/cells10071659 (PMC8304651; doi:10.3390/cells10071659)
Supplement: Supplementary file 1 [file cells-10-01659-s001.zip › cells-1261961-supplementary.pdf]

Supplementary Material

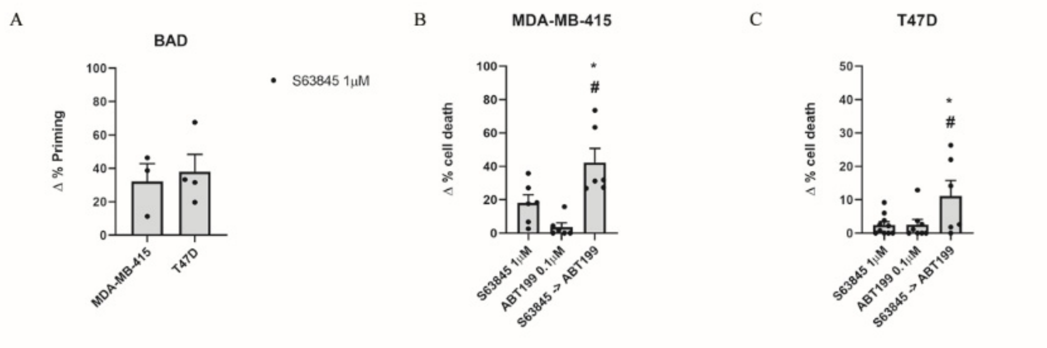

Supplementary Figure 1: BCL-2 has a mild effect mediating resistance to MCL-1 inhibition. (A) Results from the contribution of BCL-2 anti-apoptotic protein using the BAD peptide after S63845 1μM treatment in MDA-MB-415 and T47D. Results expressed as Δ% priming represents the increase in priming compared to control cells. (B-C) Cell death from Annexin V and propidium iodide staining and FACS analysis after 72 hours incubation of MDA-MB-415 and T47D cells with the single agents alone or the sequential combination of S63845 with ABT199. Values indicate mean values ± SEM. * $p<0.05$ compared to single agents and # indicates CI<1. All experiments were performed at least three times.
